# Supplementary material for: TLR2 mediates renal apoptosis in neonatal mice subjected experimentally to obstructive nephropathy
Source: PLoS One. 2023 Nov 28;18(11):e0294142. doi: 10.1371/journal.pone.0294142 (PMC10684073; doi:10.1371/journal.pone.0294142)
Supplement: S1 Raw images — Uncropped western blot gel images for TLR2 and GAPDH in neonatal WT kidneys (on day 7 and 14 of life). * marks the section used in Fig 1. TLR2 and GAPDH were visualized separately, but they represent the same gel. Uncropped western blot gel images for TLR2 and GAPDH in neonatal WT kidneys (on day 7 and 14 of life). * marks the section used in Fig 1. For Bax Gel 2 was used. Uncropped western blot gel images for Caspase-8 and GAPDH in neonatal WT and TLR2-/- kidneys (on day 7 and 14 of life). * marks the section used in Fig 2. Caspase-8 and GAPDH were visualized separately, but they represent the same gel. Uncropped western blot gel images for GSDMD and GAPDH in neonatal WT and TLR2-/- kidneys (on day 7 and 14 of life). * marks the section used in Fig 3. GSDMD and GAPDH were visualized separately, but they represent the same gel. Uncropped western blot gel images for GSDME and GAPDH in neonatal WT and TLR2-/- kidneys (on day 7 and 14 of life). * marks the section used in Fig 3. GSDME and GAPDH were visualized separately, but they represent the same gel. Uncropped western blot gel images for HMGB1 and GAPDH in neonatal WT and TLR2-/- kidneys (on day 7 and 14 of life). * marks the section used in Fig 3. Uncropped western blot gel images for TNF-α and GAPDH in neonatal WT and TLR2-/- kidneys (on day 7 and 14 of life). * marks the section used in Fig 3. TNF-α and GAPDH were visualized separately (different exposer times), but they represent the same gel. Uncropped western blot gel images for Galectin-3, TGF-β, and GAPDH in neonatal WT and TLR2-/- kidneys (on day 7 and 14 of life). * marks the section used in Fig 6. Uncropped western blot gel images for α-SMA and GAPDH in neonatal WT and TLR2-/- kidneys (on day 7 and 14 of life). * marks the section used in Fig 6. Uncropped western blot gel images for MMP-2 and GAPDH in neonatal WT and TLR2-/- kidneys (on day 7 and 14 of life). * marks the section used in Fig 6. MMP-2 and GAPDH were visualized separately, but they represen [file pone.0294142.s001.pdf]

TLR2

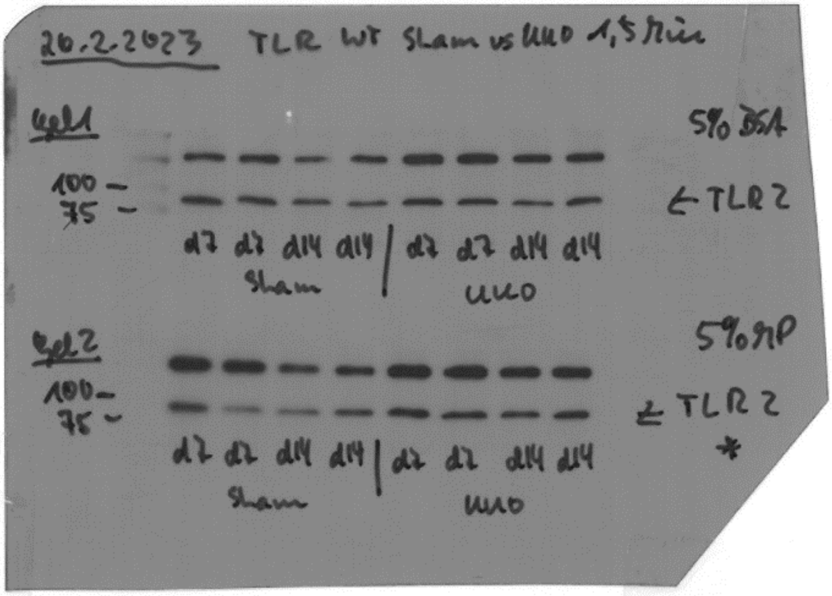
20.2.2023 TLR WT Sham vs UUO 1,5 min
gel1
5% BSA
100 –
75 –
← TLR2
d7 d7 d14 d14 d7 d7 d14 d14
Sham
UUO
gel2
5% MP
100 –
75 –
← TLR2
*
d7 d7 d14 d14 d7 d7 d14 d14
Sham
UUO

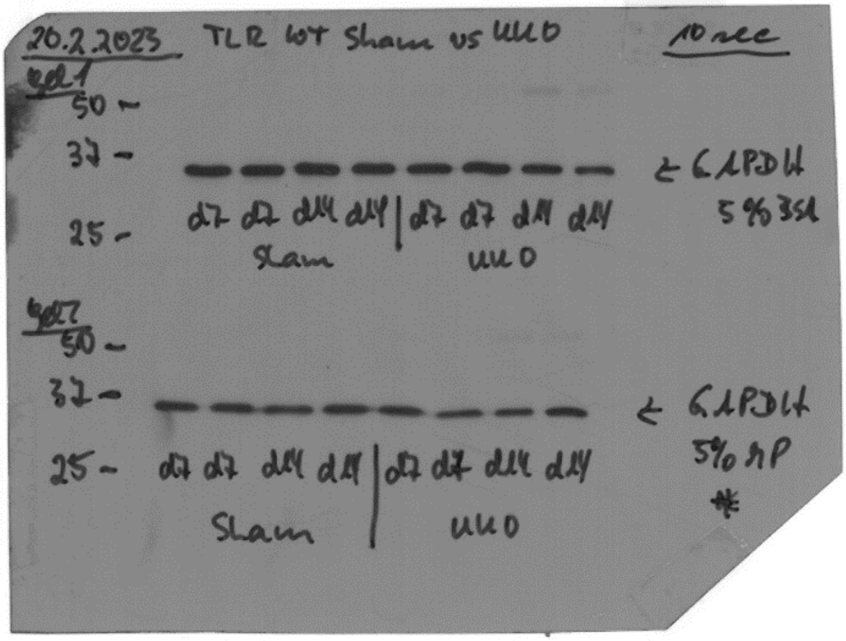
20.2.2023 TLR WT Sham vs UUO
10 sec
gel1
50 –
37 –
← GAPDH
5% BSA
25 –
d7 d7 d14 d14 d7 d7 d14 d14
Sham
UUO
gel2
50 –
37 –
← GAPDH
5% MP
*
25 –
d7 d7 d14 d14 d7 d7 d14 d14
Sham
UUO

Bcl2, Bax

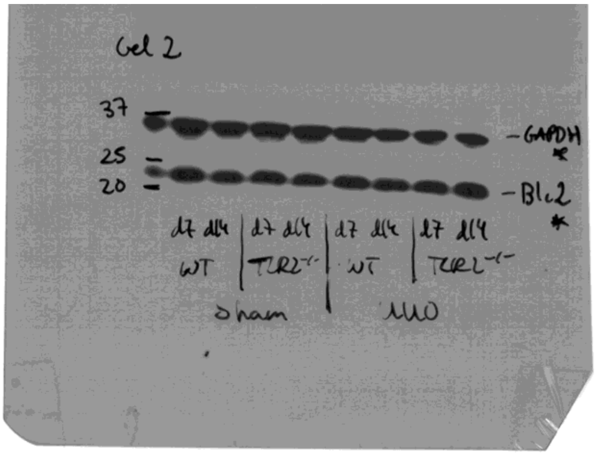
Gel 2
37
25
20
GAPDH
Blc2
d7 d14
WT
TLR2-/-
Sham
uuo

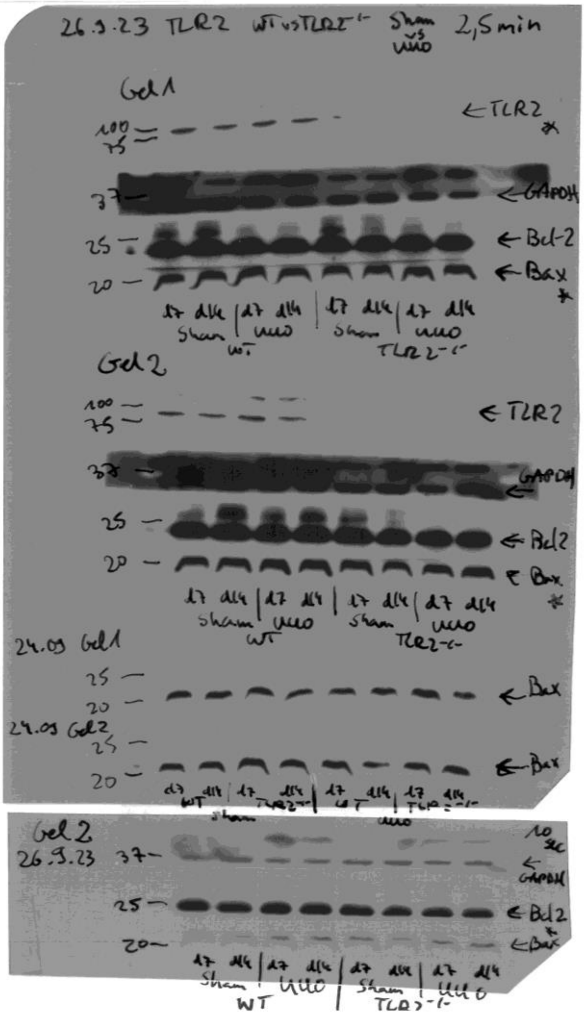
26.9.23 TLR2 WT vs TLR2-/- Sham vs uuo 2,5 min
Gel 1
TLR2
GAPDH
Bcl-2
Bax
Gel 2
TLR2
GAPDH
Bcl2
Bax
24.09 Gel 1
Bax
24.09 Gel 2
Bax
Gel 2
26.9.23
GAPDH
Bcl2
Bax
d7 d14
Sham
uuo
WT
TLR2-/-

Caspase 8

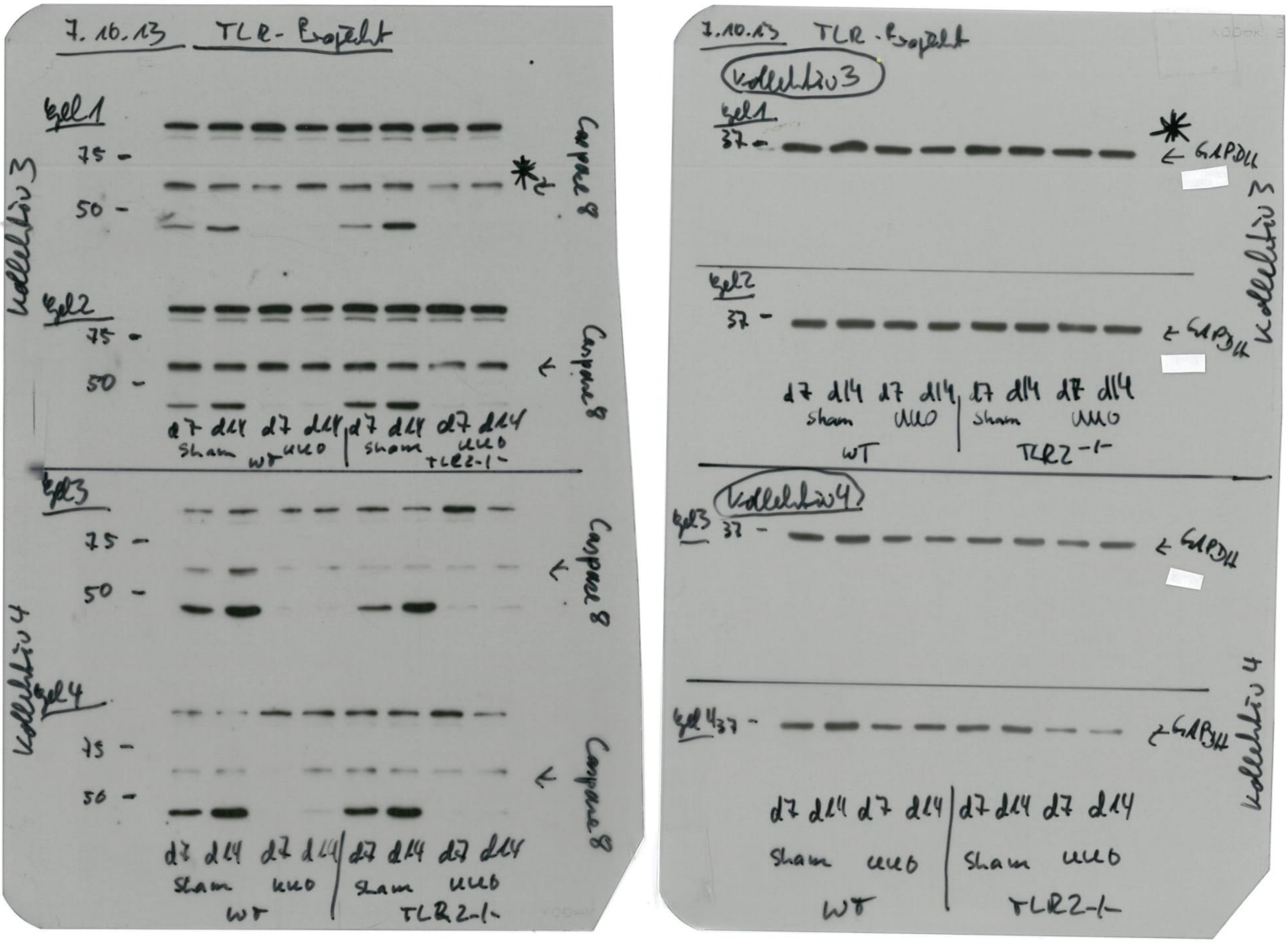
7.10.13 TLR-Projekt
Gel1
75 -
50 -
Caspase 8
Gel2
75 -
50 -
Caspase 8
d7 d14 d7 d14 d7 d14 d7 d14
Sham UUO Sham UUO
WT TLR2-/-
Gel3
75 -
50 -
Caspase 8
Gel4
75 -
50 -
Caspase 8
d7 d14 d7 d14 d7 d14 d7 d14
Sham UUO Sham UUO
WT TLR2-/-
Kollektiv 3
Kollektiv 4
7.10.13 TLR-Projekt
Kollektiv 3
Gel1
37 -
GAPDH
Gel2
37 -
GAPDH
d7 d14 d7 d14 d7 d14 d7 d14
Sham UUO Sham UUO
WT TLR2-/-
Kollektiv 4
Gel3 37 -
GAPDH
Gel4 37 -
GAPDH
d7 d14 d7 d14 d7 d14 d7 d14
Sham UUO Sham UUO
WT TLR2-/-
Kollektiv 3
Kollektiv 4

Gasdermin D

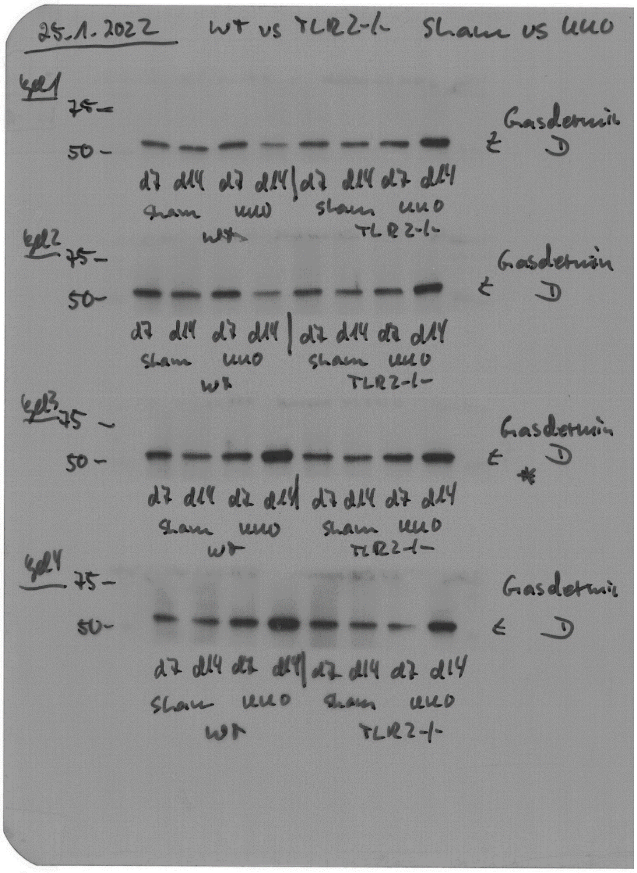
25.1.2022 WT vs TLR2-/- Sham vs UUO
gel1
75-
50-
Gasdermin D
d7 d14 d7 d14 d7 d14 d7 d14
Sham UUO Sham UUO
WT TLR2-/-
gel2
gel3
gel4

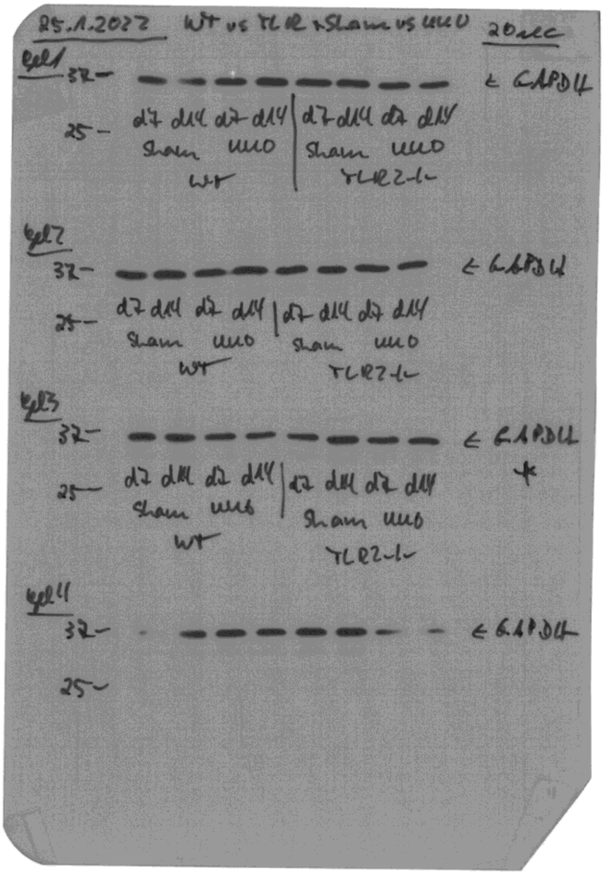
25.1.2022 WT vs TLR + Sham vs UUO
gel1
32-
25-
GAPDH
d7 d14 d7 d14 d7 d14 d7 d14
Sham UUO Sham UUO
WT TLR2-/-
gel2
gel3
gel4

Gasdermin E

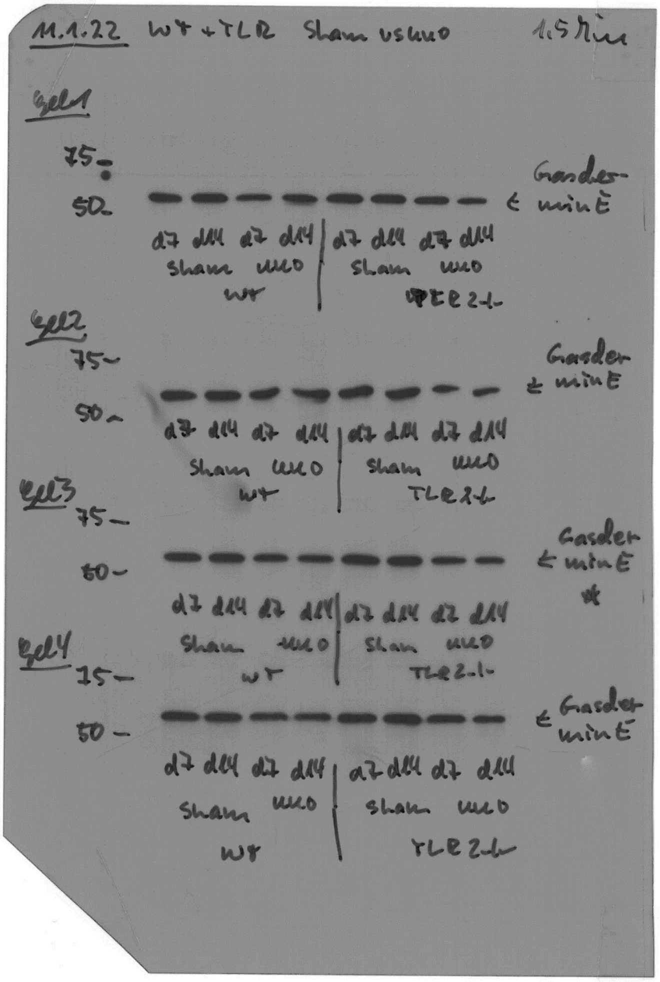

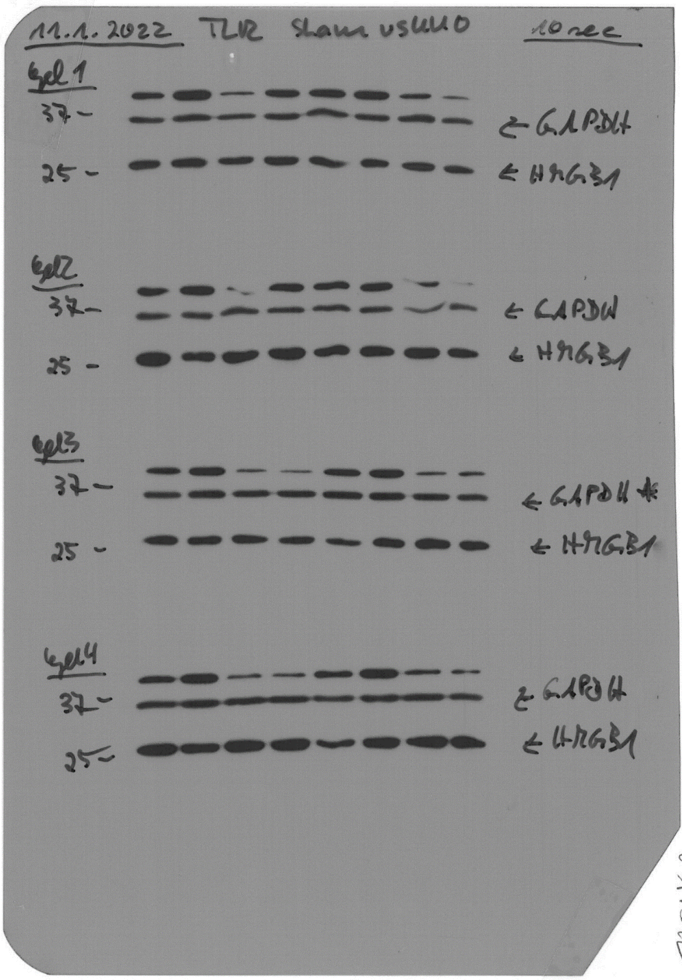

HMGB1

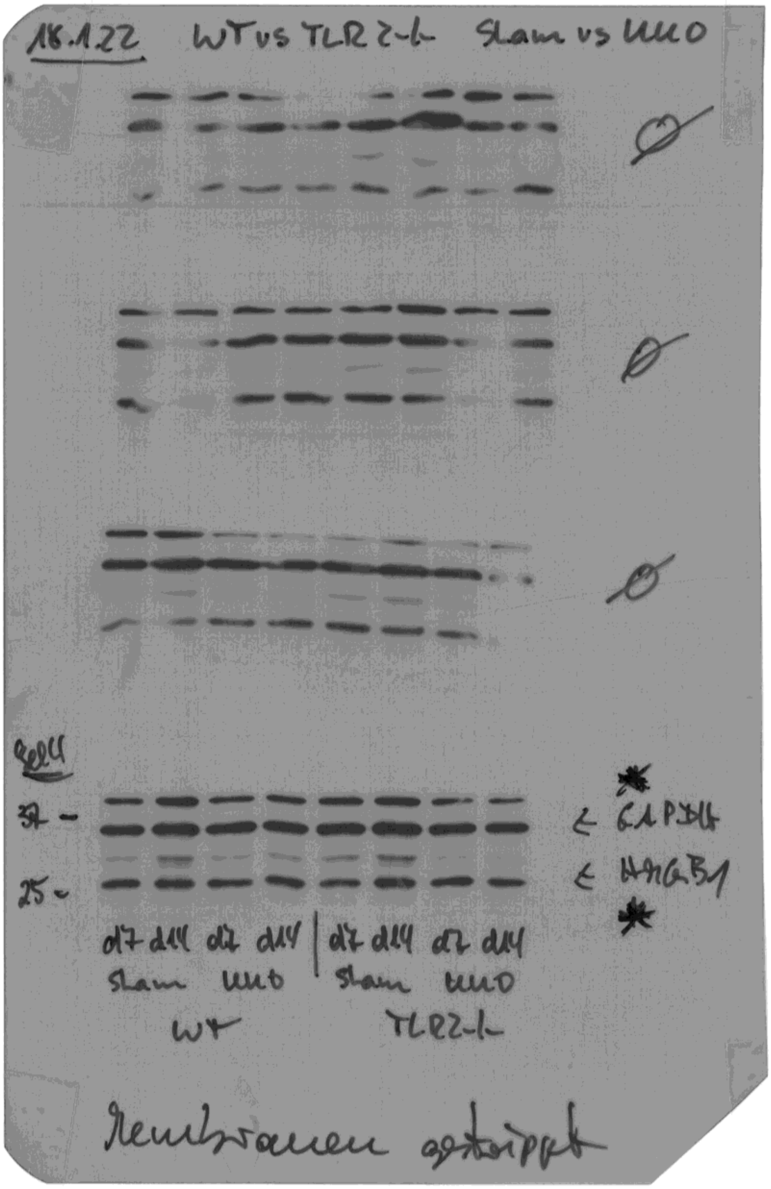
18.1.22 WT vs TLR2-/- Sham vs UUO
GAPDH
HMGB1
37
25
d7 d14 d7 d14 d7 d14 d7 d14
Sham UUO Sham UUO
WT TLR2-/-

TNF-α

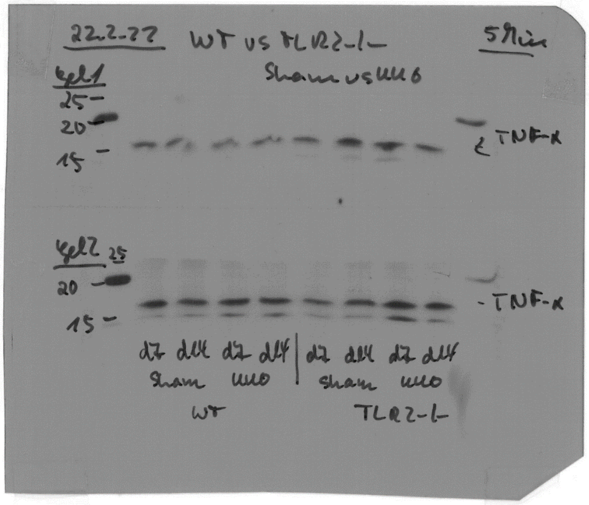
22.2.22
WT vs TLR2-/-
Sham vs UUO
5 min
gel1
25
20
15
TNF-α
gel2
25
20
15
TNF-α
d7 d14 d7 d14 | d7 d14 d7 d14
Sham UUO Sham UUO
WT TLR2-/-

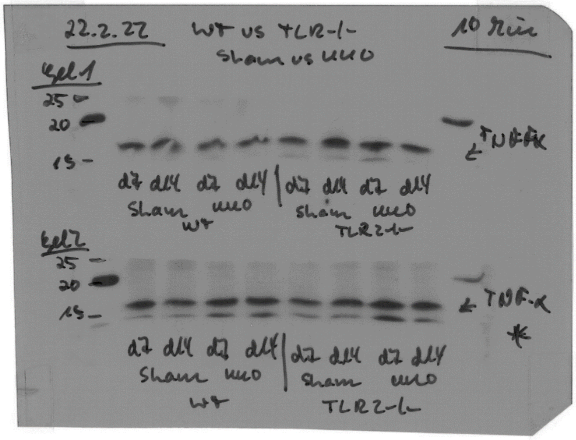
22.2.22
WT vs TLR-/-
Sham vs UUO
10 min
gel1
25
20
15
TNF-α
d7 d14 d7 d14 | d7 d14 d7 d14
Sham UUO Sham UUO
WT TLR2-/-
gel2
25
20
15
TNF-α
*
d7 d14 d7 d14 | d7 d14 d7 d14
Sham UUO Sham UUO
WT TLR2-/-

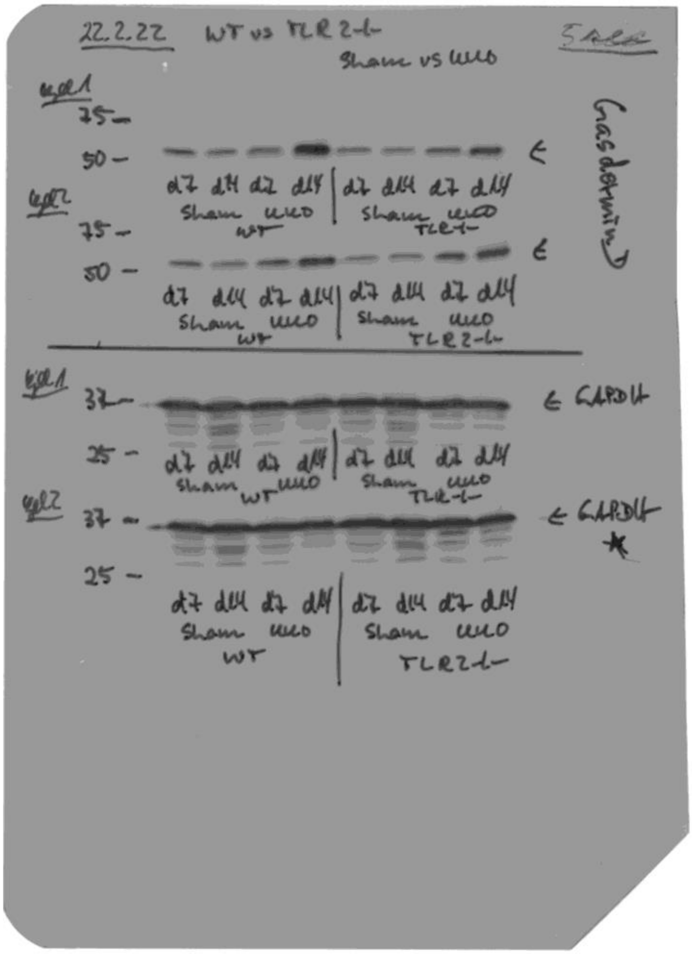
22.2.22
WT vs TLR2-/-
Sham vs UUO
gel1
75
50
Gasdermin
d7 d14 d7 d14 | d7 d14 d7 d14
Sham UUO Sham UUO
WT TLR2-/-
gel2
75
50
d7 d14 d7 d14 | d7 d14 d7 d14
Sham UUO Sham UUO
WT TLR2-/-
gel1
37
25
GAPDH
d7 d14 d7 d14 | d7 d14 d7 d14
Sham UUO Sham UUO
WT TLR2-/-
gel2
37
25
GAPDH
*
d7 d14 d7 d14 | d7 d14 d7 d14
Sham UUO Sham UUO
WT TLR2-/-

TGF-β, Galectin

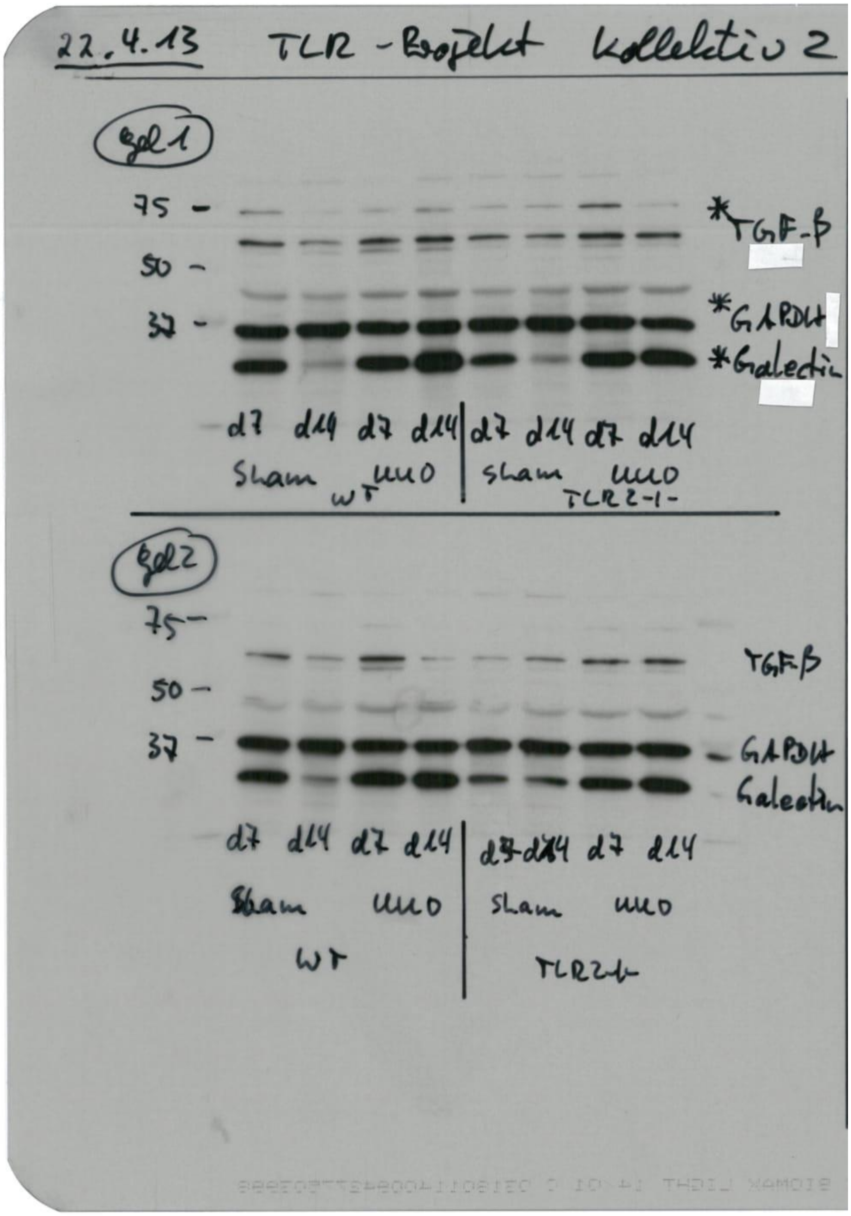

α-SMA

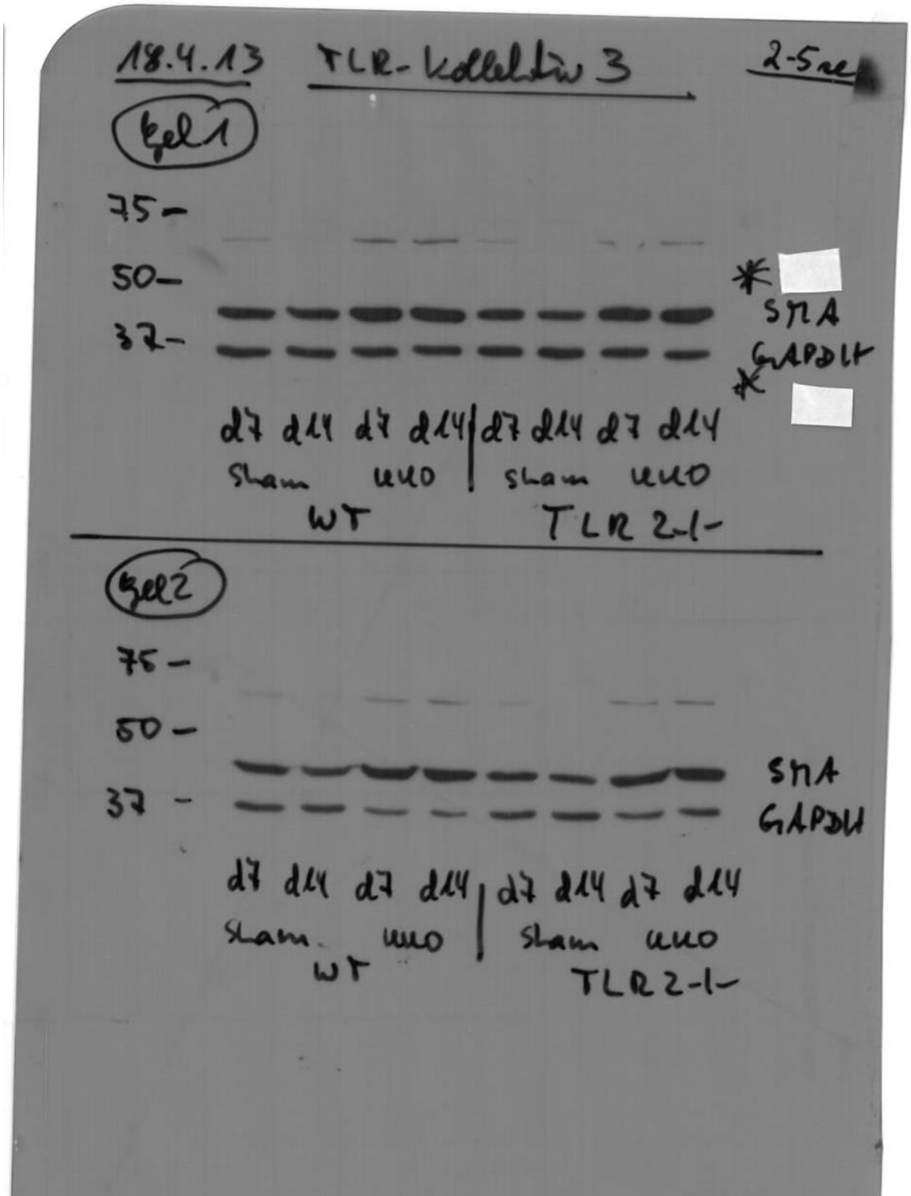
18.4.13
TLR-Kollektiv 3
Gel 1
75
50
37
SMA
GAPDH
d7 d14 d7 d14 d7 d14 d7 d14
sham UUO sham UUO
WT
TLR 2-/-
Gel 2
75
50
37
SMA
GAPDH
d7 d14 d7 d14 d7 d14 d7 d14
sham UUO sham UUO
WT
TLR 2-/-

MMP-2

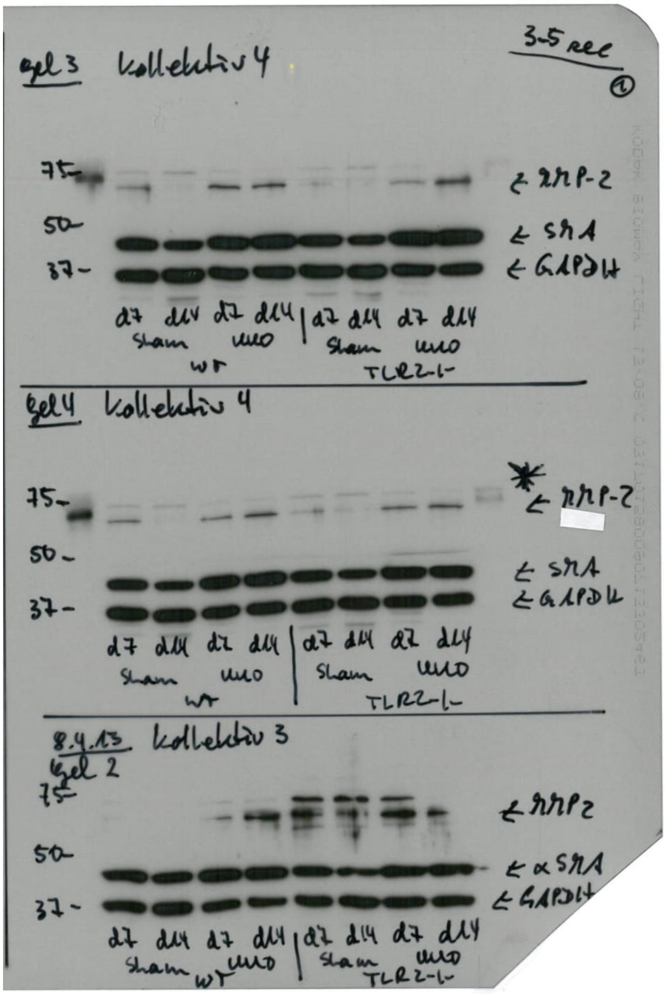
gel 3 Kollektiv 4
3-5 sec
75
50
37
← MMP-2
← SMA
← GAPDH
d7 d14 d7 d14 d7 d14 d7 d14
Sham UUO Sham UUO
WT TLR2-/-
gel 4 Kollektiv 4
← MMP-2
← SMA
← GAPDH
8.4.13 Kollektiv 3
gel 2
← MMP2
← αSMA
← GAPDH

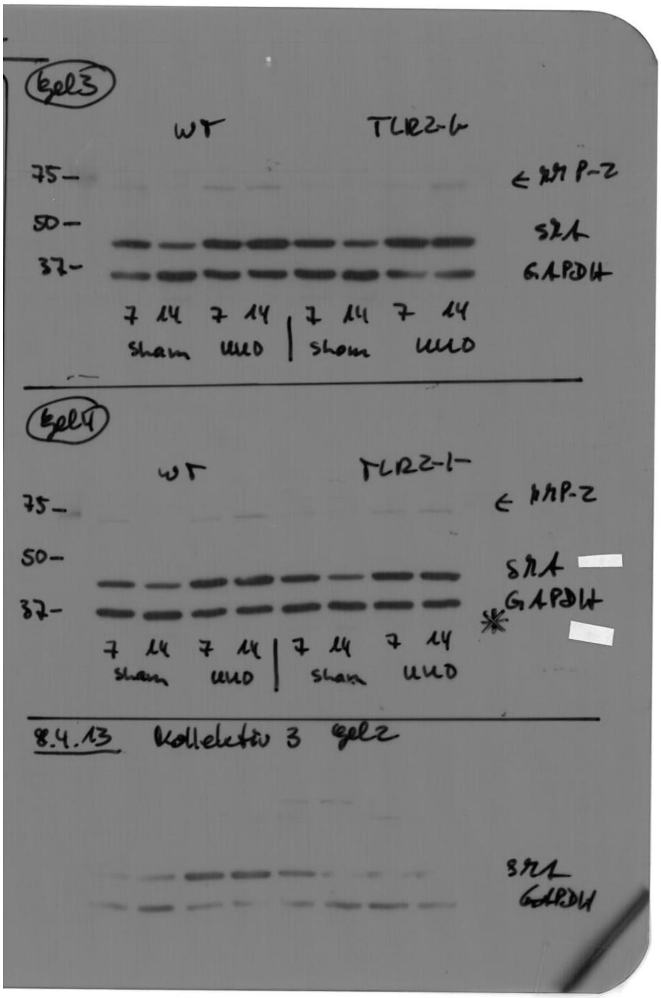
gel 3
WT TLR2-/-
75
50
37
← MMP-2
SMA
GAPDH
7 14 7 14 7 14 7 14
Sham UUO Sham UUO
gel 4
8.4.13 Kollektiv 3 gel 2
