# Supplementary material for: TLR2 mediates renal apoptosis in neonatal mice subjected experimentally to obstructive nephropathy
Source: PLoS One. 2023 Nov 28;18(11):e0294142. doi: 10.1371/journal.pone.0294142 (PMC10684073; doi:10.1371/journal.pone.0294142)
Supplement: Attachment — Submitted filename: Response to Reviewers.pdf [file pone.0294142.s003.pdf]

Klinikum der Universität München
Kinderklinik und Poliklinik im Dr. von Haunerschen Kinderspital – Innenstadt
Direktor: Prof. Dr. med. Christoph Klein

Ludwig-Maximilians-Universität München
LMU

Prof. Dr. med. Bärbel Lange-Sperandio

Klinikum der Universität München • Kinderklinik und Poliklinik im Dr. von Haunerschen Kinderspital
Lindwurmstraße 4 • D-80337 München

Pädiatrische Nephrologie

Tel. (089) 44005 2811
Fax (089) 44005 2771

Dr. von Haunersches Kinderspital

To
Prof. Dr. Franziska Theilig
Academic Editor
Plos One

München, den 30. Juni 2023

Ihr Zeichen	Unser Zeichen	Ansprechpartner	Telefon	Telefax	E-Mail
			(089) 44005 7735	(089) 44005 2771	baerbel.lange-sperandio@med.uni-muenchen.de

PONE-D-23-12099

Dear Professor Theilig,

Enclosed, please find our revised version of the manuscript entitled ‘TLR2 mediates renal apoptosis in neonatal mice subjected experimentally to obstructive nephropathy’ by Maja Wyczanska, Jana Rohling, Ursula Keller, Marcus R. Benz, Carsten Kirschning and Bärbel Lange-Sperandio.

We thank the editors and reviewers for their encouraging and insightful responses, which helped to markedly improve the manuscript. We have carefully read and addressed all the concerns raised by the referees. Please find our point-by-point response below.

We updated the methods section to include (1) efforts to alleviate suffering (see line 107) and (2) methods of sacrifice (see line 109). The section on ‘Funding Disclosure’ was changed. It reads now "The funders had no role in study design, data collection and analysis, decision to publish, or preparation of the manuscript."

The data from this study is available in a public repository (Zenodo) under this DOI: 10.5281/zenodo.8060419. The original uncropped und unadjusted images underlying blot results were submitted as ‘TLR2_uncropped gels’ with the initial submission but can also be found in the

data repository stated above.

All experiments were carried out in accordance with the Declaration of Helsinki and were approved by the institutional review boards of Ludwig-Maximilians University Munich as well as the local authorities. All authors have seen and approved of this manuscript. There is no conflict of interest for any of the authors.

We hope that you will find our revised manuscript to be suitable for publication in Plos One and very much look forward to hearing from you,

Sincerely,

B. Lange-Sperandio

Bärbel Lange-Sperandio, M.D.
Corresponding Author

Response to Reviewers

Reviewer 1

1) The subject of this manuscript is TLR2 mediates renal apoptosis in neonatal mice subjected experimentally to obstructive nephropathy. However, in this manuscript, the proportion of narrative and experimental evidence of renal apoptosis is low, while other results that do not differ are slightly more discussed, indicating a shift in emphasis in the article. Authors could increase the types of experiments on renal apoptosis or dig deeper into the mechanism of TLR2 on renal apoptosis to make full-text logic more clarity.

Ad 1) *Thank you for this important comment. We extended the section about apoptosis in our manuscript giving this topic the spotlight. You can find the additional text in lines 423-430. It reads now: 'TLR2, as a part of the innate immune system is activated by bacterial lipoproteins and signals for apoptosis through MyD88 via a pathway involving Fas-associated death domain protein and caspase-8. Inflammation following UUO is not mediated by bacteria. UUO induces sterile inflammation, which is mediated by DAMPs, of which a variety might be able to activate the TLR2 mediated apoptotic pathway. High levels of apoptosis are associated with nephron loss in the developing kidney and thus the loss of renal function.'*
We also reduced the sections in the discussion part regarding the remaining results.

2) Result I and II are both results about the center of the article, which is renal apoptosis, but they have somewhat smaller sample sizes, even less than the sample size of the undifferentiated results. It is recommended that researchers increase the sample size to make the article data more convincing.

Ad 2) *The sample sizes for immunohistochemical staining are n=8 per group for every used staining method, in the case of apoptosis it's the TUNEL staining. As for the western blot analysis, being here the analysis of caspase-8, we were only able to use 3 samples per analysis per group. Usually with adult mice it is possible to split the kidneys in parts for different analyses. However, we work with neonatal mice, of which kidney weights range on average between 15 mg at day 3 of life and only 65 mg on day 14 (sham-operated controls). The body weight of these neonatal mice also lies only between around 1.7 g on the second day of life, 4 g on day 7, and 7.3 g on day 14 of life [Lange-Sperandio B et al.* Leukocytes Induce Epithelial to Mesenchymal Transition after Unilateral Ureteral Obstruction in Neonatal Mice. *Am. J. Clin. Pathol. 2007; 861-871]. We have to perform a lysis of the whole kidney for enough material for our analyses. To ensure a large analytic range we are bound to limit our sample size used for protein analysis.*

3) The method section of the article does not describe the source of the knockout mice, and it is suggested to add a description to increase the credibility of the data. Moreover, the references of the article are old, it is recommended to use the journal literature within the last 3 years or 5 years if possible.

Ad 3) *Thank you very much for your valuable suggestions. We added the source of the knockout mice to our manuscript, see lines 102-104. It reads now: 'The $Tlr2^{-/-}$ mouse strain used (and crossed with other Tlr ko strains) has been generated by Deltagen, Cal, USA, and provided to CK through Tularik (merged into Amgen in the aftermath).'*
We also updated the literature accordingly.

Reviewer 2

1) In Figure 1, the expression of TLR2 on d14 was less than d7 in the sham group, and in the UUO group the TLR2 expression on d7 and d14 was similar. How to explain this result?
Thank you for your comments.

Ad 1) *Thank you for this important comment. We work here with neonatal mice, in which nephrogenesis is still ongoing after birth. The changes in the TLR2 expression may be developmentally differently regulated. Studies have shown that the expression of TLR2 during organ development changes with time and depends heavily on the organ [Harju K, Glumoff V, Hallman M. Ontogeny of Toll-like receptors Tlr2 and Tlr4 in mice. Pediatr Res. 2001;49(1):81-3.]. If we than combine the results of the sham-operated and UUO kidneys, we see that the increase of TLR2 following UUO on d14 is indeed higher than on d7, which is consistent with the severity of the injury.*

2) In figure 2F and 3, there were 8 groups, but histogram had only 4. What did * mean, which two groups' comparison in Figure 2E-F, Figure 3A-D, and so on? More details are needed.

Ad 2) *Thank you very much for the question. We show the results of our analyses as x-fold change above sham-operated controls. This form of presentation allows us to show the actual impact of UUO with the sham-measurements as the basis. We divide the UUO measurements by the average of the sham measurements to achieve the results we present at the end. This way we reduce the presented groups to 4 (WT vs TLR2 x-fold above sham for d7 and d14). The * displayed alone here indicates that the differences between sham and UUO are significant. We updated the figure legends for more clarification, see lines 263-264, 290-291, 316-317, 367, 376-377. It reads now: 'Standalone * represents significant differences between Sham and UUO results.'*

3) From the WB image in Figure 6B, there was no difference between d7 and d14 in UUO or Tlr2-/- group, which was different from the histogram result, and not in line with UUO progression. Besides, in the sham group, the expression of Galectin-3 on d14 was significantly reduced when compared to d7, why the expression of Galectin-3 in the sham group reduced.

Ad 3) *The differences we show here are x-fold above sham, so the decrease of α-SMA in the sham-operated controls impacts the shown results. The increase of α-SMA following UUO based on the sham results on d14 is higher than the increase on d7. This decrease of α-SMA has been shown before [Gasparitsch M et al. RAGE-mediated interstitial fibrosis in neonatal obstructive nephropathy is independent of NF-kappaB activation. Kidney Int. 2013;84(5):911-9.] in the sham-operated kidneys and is part of the ongoing nephrogenesis with mesenchymal-to-epithelial transition (MET). MET is a biological process that involves the transition from mesenchymal cells to epithelial cells as part of normal development. The drastic decrease observed in the expression of Galectin-3 is an effect of differently regulated proteins in the course of the development of the kidney. Galectin-3 plays a role in nephrogenesis [Winyard PJ et al. Epithelial galectin-3 during human nephrogenesis and childhood cystic diseases. J Am Soc Nephrol. 1997;8(11):1647-57.]. In the metanephros, the adult kidney precursor, galectin-3 was detected in the apical domains of ureteric bud branches, and there was intense expression in fetal medullary and papillary collecting ducts in both the cytoplasm and plasma membranes. Developmentally important proteins are expressed highly during nephrogenesis and decline with time, as the kidney development is gradually completed.*

4) In lines 78-80, the author described the protective effect of Tlr2-/- on inflammation and tubular injury. But the experiment results showed that Tlr2-/- had no influence on tubular inflammation, injury, or fibrosis. If it is paradox?

Ad 4) *We appreciate the comment of the referee. The protective effect of Tlr2-/- was shown in adult*

mice. However, we conducted our research on neonatal mice. We were able to show previously that neonatal mice can respond differently to UUO injury in comparison to adult mice [Kubik MJ et al. Renal developmental genes are differentially regulated after unilateral ureteral obstruction in neonatal and adult mice. Sci Rep. 2020;10(1):19302.]. We added „adult" to this text part for further clarification.

5) There was too much result explanation in the discussion of the manuscript. The experiment results showed TLR2 participated in tubular epithelial apoptosis, but had no influence on renal injury or fibrosis, neither proliferation nor inflammation. So what's the role of TLR2 in UUO induced renal injury or fibrosis? More information about TLR2 on UUO model are needed.

Ad 5) *Thank you for pointing this out. We reduced the discussion text as suggested. TLR2 seems to play a minor role in neonatal UUO regarding renal injury, fibrosis, and inflammation. It is possible that alternative or additional signaling pathways are activated in neonatal mice following UUO and the knockout of TLR2 is not effective in ameliorating the whole injury, but just apoptosis. Apoptosis itself is a non-inflammatory cell death and its inhibition does not influence inflammation [Roychowdhury S, Chiang DJ, Mandal P, McMullen MR, Liu X, Cohen JI, et al. Inhibition of apoptosis protects mice from ethanol-mediated acceleration of early markers of CCl4-induced fibrosis but not steatosis or inflammation. Alcohol Clin Exp Res. 2012;36(7):1139-47]. The inhibition of other pathways in addition of TLR2 could give promising results and help to reduce the injury done to the kidneys until obstruction is relieved.*
