# Supplementary material for: TLR2 mediates renal apoptosis in neonatal mice subjected experimentally to obstructive nephropathy
Source: PLoS One. 2023 Nov 28;18(11):e0294142. doi: 10.1371/journal.pone.0294142 (PMC10684073; doi:10.1371/journal.pone.0294142)
Supplement: Attachment — Submitted filename: 1.docx [file pone.0294142.s002.docx]

1) In Figure 1, the expression of TLR2 on d14 was less than d7 in the sham group, and in the UUO group the TLR2 expression on d7 and d14 was similar. How to explain this result?
2) In figure 2F and 3, there were 8 groups, but histogram had only 4. What did * mean, which two groups’ comparison in Figure 2E-F, Figure 3A-D, and so on? More details are needed.
3) From the WB image in Figure 6B, there was no difference between d7 and d14 in UUO or Tlr2-/- group, which was different from the histogram result, and not in line with UUO progression. Besides, in the sham group, the expression of Galectin-3 on d14 was significantly reduced when compared to d7, why the expression of Galectin-3 in the sham group reduced.
4) In lines 78-80, the author described the protective effect of Tlr2-/- on inflammation and tubular injury. But the experiment results showed that Tlr2-/- had no influence on tubular inflammation, injury, or fibrosis. If it is paradox?
5) There was too much result explanation in the discussion of the manuscript. The experiment results showed TLR2 participated in tubular epithelial apoptosis, but had no influence on renal injury or fibrosis, neither proliferation nor inflammation. So what’s the role of TLR2 in UUO induced renal injury or fibrosis? More information about TLR2 on UUO model are needed.

Review 3:

1. Please describe the experimental method of the ischemia reperfusion injury for renal fibrosis more detail. There is a big inter-procedural variation which would bias the results.

**Response:** Thank you for your comments. In our study, the model of ischemia-reperfusion was operated as below: the left renal artery of mice was clamped for 30 minutes, then clamp was removed to start reperfusion and the left kidney reverted from black to red color within approximately 10 seconds. Throughout the surgeries, animal body temperature was maintained at 36.6–37.2°C with a temperature control machine (FHC, USA). We added the description of the IR approach in details in “Methods-Mice and cells” in the revised manuscript.

2. In Figure 2, the authors exclude the possibility of direct engraftment of pEPC on reducing renal fibrosis by detecting the cell number 0.5 hour after injection. Please provide the time course results of flowcytometry within 10 days.

**Response:** We tracked infused pEPCs in operated kidney at 10 days and data was shown as below. The number of CD34 or GFP positive cells in IR+pEPCs was almost the same as in pEPCs group. We added the time course results of flowcytometry in the revised Supplementary File “Supplementary Figure 3”.

3. What is the paracrine secreting by pEPC that attentuates the pericyte transition in vitro and reduces the renal fibrosis in vivo?

**Response:** In our next plan, we collected pEPCs-cultured medium for proteomics. The relatively high-excreted factors were gathered to further explore the relationship between pEPCs and pericytes.

4. In Figure 6, the effect of p-EPC-CM on reducing TGF-beta induced pericyte transition seems less obviously than that of PDGF-BB induction. How to explain this? And why the pericyte expressing alpha-SMA before the stimulation of PDGF-BB or TGF-beta in Figure 6?

**Response:** Yes, pEPCs-CM reduced 7.9% of α-SMA in TGF-β induced transition (Figure 6B) while 17.7% in PDGF-BB induced transition (Figure 6A). We thought it may be due to the concentration of PDGF-BB in pEPCs-CM is not as high as the PDGF-BB we added into the medium. For the expression of alpha-SMA, as reported, pericyte expressed a small amount of baseline α-SMA in inactive status, but significantly up-regulated after its transition into myofibroblast [J. Clin. Invest. 2014.; Am J Pathol. 2010 Jan;176(1):85-97.].

5. What is the role of endogenous pEPC in protecting renal fibrosis following ischemia reperfusion injury?

**Response:** By parabiosis experiments, we identified that EPC does not engraft and convert into endothelial cells, which denied the direct effect of endogenous pEPCs on renal fibrosis. We supposed its protection is largely dependent on paracrine. However, as the previous report showed that, as very limited numbers in circulation, the endogenous pEPCs demonstrated less protective effect on chronic kidney diseases in clinical research [Am J Kidney Dis 2004;44:840–849.; Arterioscler Thromb Vasc Biol 2004;24:1246–1252.]. Sradnick and colleagues identified that endogenous pEPCs do not directly contribute to renal endothelial repair [J Am Soc Nephrol. 2016;27(6):1714-26]. All these data suggest that endogenous pEPCs, as the limited cell numbers, failed to help renal repair. Thus, we need to generate and expand pEPCs in vitro for the in vivo therapeutic application.

6. Please mention the way to calculate injured tubule as shown in Figure 1 in Method?

**Response:** Tubular damage included tubular dilation, tubular cell changed from cuboid to flat shape; brush border injury, loss; cast formation; tubular cell death and detachment. At least 10 high power field of view in each kidney were examined. We added the detail description in Methods “Histologic and immunofluorescent staining” in the revised manuscript.

7. What kind of EPC do you use in this research?

**Response:** The cells we injected were murine bone marrow-derived pEPCs. Bone marrow cells were harvested and seeded on fibronectin-coated plate in specific culture medium. After 7 days’ culture, cells became spindle shaped which we called early pEPCs. We did functional test and confirmed that early pEPCs were positive for Dil-Ac-LDL and UEA-1. Besides, flow cytometry showed that early pEPCs had high CD45 (85.15%), CD34 (77.14%), c-kit (88.93%) expression (Stem Cell Res Ther. 2019 Mar 21;10(1):104.) which were in accordance with previous descriptions of pEPCs (Circ Res. 2017;120(5):e7–23.).

8. In Figure 4, how to prove that the appearance of myofibroblast following injury is derived from pericytes simply by the co-staining for alpha-SMA and PDGFR-beta in the fibrotic area?

**Response:** Thanks for your comments. The co-staining for alpha-SMA and PDGFR-beta was used to detect myofibroblast deriving from PDGFR-beta positive pericytes. Except for pericytes, myofibroblasts owned other sources, such as fibroblast, tubular epithelial cells or endothelial cells etc. To accurate explore PDGFR-beta derived myofibroblast, we further tested myofibroblast marker Collagen 1α1. We added the data in Supplementary Figure 6 in the revised Supplementary file.

9. In Figure 7, is it possible that the effect of reducing renal fibrosis in DTR-PD mice is from the protection of acute ischemic injury? How about DT given after the establishment of ischemia reperfusion injury?

**Response:** Thank you for your comments. We conducted additional experiment. We gave DT 2 days after IR surgery. Compared the group “DTR-PD/IR” with “DTR-PD/IR+DT”, the former with pre-injection of DT and the latter with DT after IR, the latter showed relative increased number of injured tubules and fibrotic area. It implied us that DT can only delete existed PDGFR-β positive pericytes and prevent pericytes transition into myofibroblast. For existed myofibroblast formatted in 2 days after IR, DT can’t revert this phenomenon. We added the data in Supplementary Figure10 in the revised supplementary file.

Reviewer 4:

1. The authors should briefly describe the IR surgery. Unilateral, bilateral? Time of ischemia?

**Response:** In our study, the model of ischemia-reperfusion was operated as below: the left renal artery of mice was clamped for 30 minutes, then clamp was removed to start reperfusion and the left kidney reverted from black to red color within approximately 10 seconds. Throughout the surgeries, animal body temperature was maintained at 36.6–37.2°C with a temperature control machine (FHC, USA). We added the description of the IR approach in details in “Methods-Mice and cells” in the revised manuscript.

.

2. Where is the BUN data

**Response:** In supplementary file. Supplementary Figure 5B.

3. Was only cortex evaluated? What about medulla? Were they compared?

**Response:** We evaluated the entire field of vision, including both cortex and medulla. For pathological staining, we calculated 200x visual field to evaluate renal injury and fibrosis. The glomerulus in staining was to ensure the same physiological location among different groups and correction bias.

4. Figure 1C: Authors should be more specific what left kidney/right kidney means. Maybe add an axis. a. The percentage of interstitial fibrosis is extremely low. Is the axis correct?

**Response:** Thanks for the comments. We corrected “left kidney/right kidney” to “left kidney/right kidney weight”. “Tubular injury and interstitial fibrosis implied attenuated chronic renal damage. And the “left kidney/right kidney weight” was adopted to evaluate renal fibrosis as when fibrosis progressed, parenchymal cells (tubular epithelial cells, podocytes, etc.) in situ loss and fibrotic collagens deposition, which led to weight loss of injured kidney and the decrease of left kidney/right kidney weight” ratio (Figure 1C).”

**Response:** As for the low percentage of interstitial fibrosis, it may be attribute to the moderate model of IR and the adopted measurement with MASSON staining, which is not as sensitive as immunofluorescent staining.

5. You mention in the text that “pEPCSs preserved the density and structure of interstial capillaries (Figure 2A). They didn’t preserve the density but attenuated or minimized density loss.

**Response:** Thanks for your suggestion. We corrected “preserved the density and structure of interstitial capillaries” to “attenuated density loss”.

6. Figure 2C: I think as a supplement, the authors should show the gating strategy and images from cytometer or analysis software. -May not need this as there was no difference.

**Response:** Thanks for your suggestion. We showed the gating strategy and images in Supplementary Figure 3A.

7. Figure 4 D: I need some clarification. It appears there is substantial proliferation in the Cm group, but reduced in the bar graph. Please explain.

**Response:** We calculated only the number of proliferative pericytes (areas outside dotted frames were renal interstitium where pericytes located). In the pEPCs-CM group, most of the PCNA positive cells were tubular epithelial cells (cells inside dotted frame were tubular epithelial cells).
