# Supplementary material for: TLR2 mediates renal apoptosis in neonatal mice subjected experimentally to obstructive nephropathy
Source: PLoS One. 2023 Nov 28;18(11):e0294142. doi: 10.1371/journal.pone.0294142 (PMC10684073; doi:10.1371/journal.pone.0294142)
Supplement: Attachment — Submitted filename: Response to Reviewers.doc [file pone.0294142.s004.doc]

| Klinikum der Universität München      Kinderklinik und Poliklinik im Dr. von Haunerschen Kinderspital – Innenstadt  Direktor: Prof. Dr. med. Christoph Klein  **Prof. Dr. med. Bärbel Lange-Sperandio** | |  |  |
| --- | --- | --- | --- |
| Klinikum der Universität München • Kinderklinik und Poliklinik  im Dr. von Haunerschen Kinderspital  Lindwurmstraße 4 • D-80337 München |  |  | |
| To  Prof. Dr. Franziska Theilig  Academic Editor  Plos One |  | Pädiatrische Nephrologie  Tel. (089) 44005 2811 Fax (089) 44005 2771 | |
|  |  | München, den 30. November 2026 | |

Ihr Zeichen	Unser Zeichen	Ansprechpartner	Telefon	Telefax	E-Mail

(089) 44005 7735	(089) 44005 2771	baerbel.lange-sperandio@med.uni-muenchen.de

PONE-D-23-12099R1

Dear Professor Theilig,

Enclosed, please find our revised version of the manuscript entitled ‘TLR2 mediates renal apoptosis in neonatal mice subjected experimentally to obstructive nephropathy’ by Maja Wyczanska, Jana Rohling, Ursula Keller, Marcus R. Benz, Carsten Kirschning and Bärbel Lange-Sperandio.

We thank the editors and reviewers for their encouraging and insightful responses, which helped to markedly improve the manuscript. We have carefully read and addressed all the concerns raised by the referees including additional experiments on Bcl-2 and Bax expression. Please find our point-by-point response below.

We hope that you will find our revised manuscript to be suitable for publication in Plos One and very much look forward to hearing from you,

Sincerely,

Bärbel Lange-Sperandio, M.D.

Corresponding Author

**Response to Reviewers**

**Reviewer 1**

**1)** In result 1“Neonatal UUO induces protein expression of TLR2”, authors only showed the results of Western Blotting experiment, I suggest that authors can add the pathological results of the sham group versus the UUO group, moreover, authors are able to testify the indicators of renal fibrosis.

**Ad 1)** *Thank you for this valuable comment. We absolutely agree that it is important to show the pathological results of UUO. The pathological effects of neonatal UUO are thoroughly studied [1]. We indicate these changes in our study as well. The standalone asterisks (*) in the bar plots indicate that the results shown were significantly different between the sham and UUO group. This way we are able to show the overall pathology of neonatal UUO and the differences between Tlr2^-/-^ and WT mice in one plot.*

**2)** In result 2 “TLR2 mediates tubular and interstitial apoptosis in neonatal kidneys with UUO”; Although authors used the knockout mouse, did not demonstrate the evidence of decreasing the expression of TLR2. On the other hand, the indicators of Western Blotting verifying apoptosis are insufficient, authors can add Bax and Bcl-2 etc.

# Ad 2) *Thank you for this important suggestion. We agree that evidence that the knock-out mice indeed do not express TLR2 is essential. We added to figure 1 the results of a western blot analysis showing, that in comparison to WT the Tlr2^-/-^ mice do not express TLR2 protein. We also added a text to the results section regarding this analysis, you can find it in lines 224-225 and it reads: “This analysis was also used to confirm that Tlr2^-/-^ mice indeed did not express TLR2 (Fig 1B).”*

*Thank you for the comment regarding additional apoptosis experiments. We performed additional western blots of Bcl-2 and Bax. The protein expression of Bcl2 after ureteral obstruction increased significantly in Tlr2^-/-^ mice at day 14 of life in comparison to WT. As Bcl-2 is known to suppress apoptosis [2], this confirms our findings, that TLR2 mediates apoptosis in the UUO model and that Tlr2^-/-^ kidneys show less apoptosis after neonatal UUO in comparison to WT. We also measured the protein expression of Bax, an apoptosis activator [2] in neonatal mice kidneys. There was no significant difference in Bax expression between Tlr2^-/-^ and WT mice. We added the results of both analyses to figure 3 and included these in the results and discussion sections in lines 259-264 and 441-443. It reads now: “For further analysis of cell death in our model we analyzed the anti-apoptotic marker Bcl-2 and the pro-apoptotic marker Bax using western blot (Fig 3). Neonatal Tlr2^-/-^ mice showed a higher expression of Bcl-2 at day 14 in comparison to WT (Fig 3A), confirming that TLR2 mediates apoptosis in the neonatal model of obstructive nephropathy. Bax expression increased following UUO at day 14, without significant differences between Tlr2^-/-^ and WT kidneys.” And (discussion) “As our results were conflicting, we decided to include Bcl-2 as an anti-apoptotic marker [2, 3]. The increase of Bcl-2 expression in Tlr2^-/-^ kidneys further confirms our findings that TLR2 mediates apoptosis in neonatal UUO.”.*

**3)** The results of subsequent experiments by the author were all negative, however, displaying experiments were inadequate to demonstrate the regulation of TLR2 on renal apoptosis. I recommend authors to supplement experiments to make the paper more logical and compact.

Ad 3) *Thank you for this valuable insight. We agree that the displayed results were not strong enough to show that TLR2 mediates apoptosis in the neonatal UUO model. We hope that the additional experiments we performed are sufficient to confirm our findings in this study. We think that these results have important implications for future research on TLR2.*

1.	Chevalier, R.L., M.S. Forbes, and B.A. Thornhill, *Ureteral obstruction as a model of renal interstitial fibrosis and obstructive nephropathy.* Kidney Int, 2009. **75**(11): p. 1145-1152.

2.	Chipuk, J.E., et al., *The BCL-2 family reunion.* Mol Cell, 2010. **37**(3): p. 299-310.

3.	Zhang, G., et al., *Role of apoptosis and Bcl-2/Bax in the development of tubulointerstitial fibrosis during experimental obstructive nephropathy.* Exp Nephrol, 2001. **9**(2): p. 71-80.
